# Nonurgent patients in the emergency department? A French formula to prevent misuse

**DOI:** 10.1186/1472-6963-10-66

**Published:** 2010-03-15

**Authors:** Stéphanie Gentile, Pascal Vignally, Anne-Claire Durand, Sabina Gainotti, Roland Sambuc, Patrick Gerbeaux

**Affiliations:** 1 Department of Public Health, Faculty of Medicine, Marseille, France; 2 Emergency Department of University Hospital, Marseille, France

## Abstract

**Background:**

Overcrowding in emergency department (EDs) is partly due to the use of EDs by nonurgent patients. In France, the authorities responded to the problem by creating primary care units (PCUs): alternative structures located near hospitals. The aims of the study were to assess the willingness of nonurgent patients to be reoriented to a PCU and to collect the reasons that prompted them to accept or refuse.

**Methods:**

We carried out a cross sectional survey on patients' use of EDs. The study was conducted in a French hospital ED. Patients were interviewed about their use of health services, ED visits, referrals, activities of daily living, and insurance coverage status. Patients' medical data were also collected.

**Results:**

85 patients considered nonurgent by a triage nurse were asked to respond to a questionnaire. Sex ratio was 1.4; mean age was 36.3 +/- 11.7 years.

Most patients went to the ED autonomously (76%); one third (31.8%) had consulted a physician. The main reasons for using the ED were difficulty to get an appointment with a general practitioner (22.3%), feelings of pain (68.5%), and the availability of medical services in the ED, like imaging, laboratory tests, and drug prescriptions (37.6%). Traumatisms and wounds were the main medical reasons for going to the ED (43.5%).

More than two-thirds of responders (68%) were willing to be reoriented towards PCUs. In the multivariate analysis, only employment and the level of urgency perceived by the patient were associated with the willingness to accept reorientation. Employed persons were 4.5 times more likely to accept reorientation (OR = 4.5 CI (1.6-12.9)). Inversely, persons who perceived a high level of urgency were the least likely to accept reorientation (OR = 0.9 CI (0.8-0.9).

**Conclusions:**

Our study provides information on the willingness of ED patients to accept reorientation and shows the limits of its feasibility. Alternative structures such as PCUs near the ED seem to respond appropriately to the growing demands of nonurgent patients. Reorientation, however, will be successful only if the new structures adapt their opening hours to the needs of nonurgent patients and if their physicians can perform specific technical skills.

## Background

For several decades, French hospitals have faced overcrowding in Emergency Departments (EDs). This phenomenon is mostly due to a misuse of EDs on the part of patients who use EDs for nonurgent problems [1-16]. In fact, patients requiring vital interventions represent less than 3% of those using EDs [11,12]. Overcrowding in EDs is described in the Emergency Medicine literature as a major public health problem because of its consequences: degradation of the quality of care (prolonged waiting times, delays to diagnosis and treatment, delays in treating seriously ill patients), increased costs (leading to unnecessary diagnostic investigations), and patients' dissatisfaction [13,17]. Nonurgent patients' use of EDs, rather than primary care settings, provides the opportunity to be cured without an appointment, in a place that has modern and high quality technologies [11,14,15,18]. Moreover, ED patients often report that general practitioners (GPs) are not available at nights and on weekends [11,18-20].

In this context, the French government implemented several measures to improve the coordination of health care services and EDs and to control the flow of ED visits [21]. One of the measures was to develop alternative health care structures named primary care units (PCU). These PCUs may be localized in the hospital near EDs, which facilitates the transition of patients from one service to another. However, this localization is still marginal [11,22]. PCUs may also be outside the hospital and are termed GP consultations without appointment, but they require the patient to leave the hospital [22-24]. These alternative health care structures, can take care of nonurgent patients who go by themselves to an ED or have been wrongly directed to one.

The reorientation of nonurgent patients to a PCU must be decided in the triage area of the ED [24,25]. However, the reorientation presents medico-legal problems if it is performed by a non-physician. But, in fact, few ED physicians are available in the triage area, due to the costs of this provision [26]. In most cases, the reorientation is performed by a triage nurse following the guidelines of the French Society of Medical Emergency. The triage nurse must assess the patients' needs for care and the severity of their health problems in order to reorient them in a timely and adequate manner. If a PCU is available near the ED, the triage nurse may suggest (but cannot require) that patients be examined at the PCU for their health problems [27].

We conducted a study in the ED of a University Hospital of Marseille to assess the willingness of nonurgent patients to be reoriented outside the ED to a PCU. The secondary objective was to collect the reasons that prompted them to accept or refuse. The results of this study will inform policy makers on the acceptability of PCUs.

## Methods

### Study setting

A cross-sectional study was conducted in the adult ED of La Conception Hospital in Marseille, France. Data was collected during a week, from 9 am to 8 pm. Each day, two time slots of two hours corresponding to peak ED consultation were randomly selected.

### Population

We included all patients aged 18 years and older presenting to the ED during the study period, arriving by their own means and identified as nonurgent by a triage nurse in the triage area. Consent for an interview and medical chart access was obtained from the patients. Confidentiality was ensured. Patients were excluded if they had communication difficulties not related to their presenting complaint.

### Study protocol

Data were collected immediately after the triage process and before examination by the medical staff of the ED. Following informed consent, patients were invited to complete a questionnaire with a research assistant in the ED. The standardized questionnaire included 32 items [Additional file 1] The first part of the questionnaire covered socio-demographic data (age, gender, country of origin, marital status) and socio-economic characteristics (level of education, employment status, health insurance status); the second part covered the patient's usual source of care (follawed by a GP, number of GP consultations in the last year); the third part covered the ED visit (day for visiting the ED, principal reason for attending the ED, duration of presenting problem, previous contact with a physician for the same reason prompting the patient to visit the ED, reference to the ED, level of urgency perceived by the patient on a scale from 0 for "no urgency" to 20 for "extremely urgent problem").

A last part was designed to assess the willingness of patients identified as nonurgent to be reoriented to a hypothetical PCU outside the ED and to explore factors associated or not with this reorientation (such as guarantee to be received in consultation, a PCU near the hospital, and request for an ED physician's agreement for the reorientation...).

At the end of the ED visit, a short questionnaire was completed by ED physicians for each patient included [Additional file 2]. The variables covered investigations and treatments performed in the ED, and referral and discharge decisions made (home or hospital admission).

### Data analysis

Data were collected on Microsoft Excel software and analyzed on Spss 15.0. We compared all the variables characterizing the patient and the ED visit with the patient's willingness to accept or not the reorientation to a PCU. Data were analyzed by using frequencies and χ^2 ^tests to investigate the association between dichotomous and categorical variables. The Mann-Whitney U test was used for ordinal variables and Student's t test for continuous variables.

Moreover, we used a multivariate logistic regression method to quantify the strength of the independent associations between factors linked to the patient and the willingness regarding reorientation to a PCU. Variables obtaining a p value < 0.2 in the univariate analysis were included in the logistic regression analysis.

Our study does not need to be approved by an ethics committee under the criteria of the bioethics law. Indeed, the transposition into French law of Directive 2001/20/CE, which relates to good clinical practice in the conduction of trials on drugs for human use, has required the modification of certain provisions that concern the protection of persons participating in biomedical research, in particular those provisions concerning the conditions for the authorization of biomedical research. The law 2004-806 of August 9, 2004 categorize three research categories: biomedical research, research on standard care, and non interventional research [28]. The non interventional research does not need to be approved by an Committee for the Protection of Persons, because of its observational nature and does not require the authorization of the National Commission for Informatics and Freedom due respect for patient anonymity.

## Results

During the study period, 630 consecutive adult patients visited the ED. Among them, 245 (38.9%) came in the time slots randomly. Among these 245 patients, triage nurses identified 110 patients as nonurgent, that is, who could be treated by a GP (44.9%); 85 of them were interviewed (77.2%). Twenty-five patients were excluded from the analysis because they left without being seen, refused to participate in the study, or were unable to provide data because of altered mental status or other reasons.

### Characteristics of ED patients identified as nonurgent by triage nurses

The mean age of nonurgent patients was 36.3 years (SD, 11.7; range 18-70). In this group, 58.8% were men and 69.4% were employed [Table 1]. Most of the nonurgent patients had medical insurance (80%); 23.5% of them were covered by French national health insurance for individuals and families with low income and resources. This insurance is called the "CMU". The majority of nonurgent patients reported being followed by a GP (70.6%) [Table 2].

**Table 1:** Characteristics of ED patients identified as nonurgent willing to accept reorientation

	Accepting n = 58	Not accepting n = 27	Total n = 85	p
Mean age ± SD**	36.1 ± 12.7	36.6 ± 9.3	36.3 ± 11.7	0.858

	n (%*)	n (%*)	n	

Sex				0.373
Male	36 (62.1)	14 (51.9)	50	
Female	22 (37.9)	13 (48.1)	35	
Birthplace				0.124
Other country	16 (27.6)	12 (44.4)	28	
France	42 (72.4)	15 (55.6)	57	
Marital status				0.682
Living without a partner	13 (22.4)	5 (18.5)	18	
Living with a partner	45 (77.6)	22 (81.5)	67	
Level of education				
More than basic education	26 (44.8)	8 (29.6)	34	0.183
Basic education or less	32 (55.2)	19 (70.4)	51	
Employment status				0.004
Employed	46 (79.3)	13 (48.1)	59	
Unemployed	12 (20.7)	14 (51.9)	26	

* Column percentages, ** SD: Standard Deviation

**Table 2:** Medical insurance and regular source of care

	Accepting n = 58	Not accepting n = 27	Total n = 85	p
	n (%*)	n (%*)	**n**	

Medical insurance with supplementary health insurance				1.000
Yes	48 (82.8)	23 (85.2)	71	
No	10 (17.2)	4 (14.8)	14	
CMU**				0.002
Yes	8 (13.8)	12 (44.4)	20	
No	50 (86.2)	15 (55.6)	65	
Regular source of care				0.588
Yes	42 (72.4)	18 (66.7)	60	
No	16 (27.6)	9 (33.3)	25	
Source of care				0.127
Followed by a GP	36 (62.1)	12 (44.4)	48	
Other	22 (37.9)	15 (55.6)	37	
Mean of consultations at the GP during the last year (± SD***)				
	2.9 ± 3.1	4.2 ± 5.1	3.3 ± 3.9	0.170
ED visits during the last year				0.065
None	40 (69.0)	13 (48.1)	53	
One time or more	18 (31.0)	14 (51.9)	32	

* Column percentages, ** CMU: French health insurance designed specifically to individuals and families with low incomes and resources, *** SD: Standard Deviation

### Circumstances and reasons provided for attending the ED

Presenting problems had lasted less than 24 hours for two third of patients identified as nonurgent by triage nurses. One third of nonurgent patients had tried to contact their GP before presenting to the ED. Most nonurgent patients were self-referred (76%); the others were referred by their GP (17.6%) or referred for medico-legal reasons (5.9%) (employer, police) [Table 3].

**Table 3:** Circumstances and reasons provided for attending the ED

	Accepting n = 58	Not accepting n = 27	Total n = 85	p
	n (%*)	n (%*)	**n**	

Day of ED visit				0.277
Weekday	36 (62.1)	20 (74.1)	56	
Weekend day	22 (37.9)	7 (25.9)	29	
Previous contact with a GP				0.225
Yes	16 (27.6)	11 (40.7)	27	
No	42 (72.4)	16 (59.3)	58	
Duration of presenting problem				0.941
≤ 1 day	37 (63.8)	17 (63.0)	54	
> 1 day	21 (36.2)	10 (37.0)	31	
Referral to the ED				0.664
Self/Relative referral	46 (79.3)	19 (70.4)	65	
GP	9 (15.5)	6 (22.2)	15	
Employer	3 (5.2)	2 (7.4)	5	
Mean level of urgency perceived by the patient (± SD**)	9.4 ± 5.5	13.0 ± 5.2	10.6 ± 5.6	
Pain				0.577
Yes	38 (65.5)	16 (59.3)	54	
No	20 (34.5)	11 (40.7)	31	
Principal reason for attending the ED (n = 83)				0.100
Somatic injuries	24 (42.1)	16 (61.5)	40	
Traumatic injuries	33 (57.9)	10 (38.5)	43	
Diagnostic investigation(s) performed at the ED				0.348
Yes	21 (36.2)	7 (25.9)	28	
No	37 (63.8)	20 (74.1)	57	
Treatment for trauma performed at the ED				1.000
Yes	4 (6.9)	2 (7.4)	6	
No	54 (93.1)	25 (92.6)	79	

* Column percentages, ** SD: standard deviation

The most common reasons provided for attending the ED were pain (65.8%) and a need for diagnostic investigations (37.6%). Half the nonurgent patients were consulting for traumatologic problems. Nearly one quarter (22.3%) came to the ED because of difficulty in accessing their usual sources of care.

The mean level of urgency perceived by the patient was 10.6 ± 5.6 (median = 10; range 1-20). One third received additional investigations; only 6 patients received treatment, and none were hospitalized.

### Nonurgent patients' willingness to accept or not the reorientation to a PCU

More than two-thirds of patients identified as nonurgent (68.2%) would accept being reoriented to a PCU by the triage nurse. Patients accepting the reorientation were significantly more likely to be employed, benefited less from "CMU" insurance [Table 2], and had a lower mean level of perceived urgency [Table 3]. In the multivariate analysis, willingness to accept reorientation was associated with employment status and the level of urgency perceived by the patient [Table 4]. Employed persons were 4.5 times more likely to accept reorientation (odds ratio (OR), 4.5; 95% confidence interval (CI), 1.6-12.9). Inversely, persons who perceived a high level of urgency were the least likely to accept a reorientation (OR, 0.9; 95% CI, 0.8-0.9).

**Table 4:** Variables associated with willingness to accept reorientation

		Odds ratio and 95% confidence interval	p
Level of urgency perceived by the patient	13 ± 5.2	0.9 (0.8-0.9)	0.019

Employed	12 (22.0%)	4.5 (1.6-12.9)	0.005

Thirteen percent of nonurgent patients willing to accept reorientation did not want to go to a PCU located outside the hospital. Several nonurgent patients agreeing to be reoriented to a PCU outside the hospital stipulated certain conditions: a guarantee to be received in consultation (23.5%), a PCU near the hospital (23.5%), and the agreement of an ED physician for the reorientation (17.6%).

Among the nonurgent patients refusing reorientation (31.8%), almost 41% would be willing to pay an extra fee for being treated at the ED. These patients had a higher level of education (p = 0.038).

## Discussion

This study describing the use of ED by nonurgent patients and the willingness to accept reorientation to PCU is based on a small sample size. However, the sample seems representative of the target population because of the following reasons. The socio-demographic characteristics of our sample were similar to those reported in the literature on the subject [14,15,29-31]: included patients were young, male, with a low level of education, and employed. As in the literature, most patients decided to go to the ED directly without prior contact with their GP and pain perceived by the patient, need of access to diagnostic investigations, and difficulty in accessing ambulatory healthcare services were the most common reasons provided by patients for attending the ED [12-14,29-31].

However, our sample has two specificities. First, they had a more precarious socio-economic status than the general French population. Indeed, a high percentage of patients had "CMU" insurance (23.5% in our study versus 8% for the general French population) and a high percentage had complementary insurance (16.5% in our study versus 7% for the general French population) [32]. Second, the proportion of nonurgent patients identified by the triage nurse for reorientation (44.9%) may seem overestimated compared to proportions in other publications (30%) [13,14,33-37]. The proportion of nonurgent patients found in the literature varies considerably (4.8% to 90%) according to the triage criteria used and time of categorization into urgent or nonurgent cases (prospectively in triage area or retrospectively at the end of the consultation). However, none of nonurgent patients included in our study were hospitalized, which confirms the validity of our sample.

Our results show that nonurgent patients are willing to accept potential reorientation to a PCU. The willingness to accept reorientation appears to be related to a higher economic level; also, the patients accepting the principle were more likely to be employed. The perceived level of urgency is also a key element, which confirms the need to educate patients about which problems require care at the ED and those that require an consultation with a GP [7,11,18,36-38]. However, willingness to accept reorientation depended on the following prerequisite: the guarantee of obtaining a medical consultation in a PCU located near the ED. To ensure this prerequisite and properly manage patient flow, the PCU and EDs will have to cooperate and coordinate their activities. Moreover, we note that nearly 58% of patients accepting reorientation consulted for traumatic problems, which requires as a prerequisite the ability of GPs to practice acts of minor surgery (such as sutures and strapping).

Our study highlights another important result: nearly one third of patients refusing reorientation would be willing to pay an extra fee to be treated in the ED. This result requires consideration because it shows the actual motivation of these patients in their choices to go to EDs.

Altogether, results show ED patients agree to be reoriented to a PCU. PCUs seem to be a relevant solution to solve the problem of ED overcrowding [24,25]. In practice, the implementation of PCUs depends on the proximity of the structure, the conformity of time slots between EDs and PCUs, and the realization of acts of minor surgery.

## Conclusion

Our study provides essential information on the willingness of ED patients identified as nonurgent to accept reorientation to an alternative health care structure and shows the limits of its feasibility. Alternative structures such as primary care units near the ED seem to be respond appropriately to the growing demands of nonurgent patients.

## Competing interests

The authors declare that they have no competing interests.

## Authors' contributions

SG participated in the design and the coordination of the study, performed the statistical analysis and helped to draft the manuscript. PV, SG, ACD and PG participated in the design of the study, interpreted the results and helped to draft the manuscript. RS participated in the statistical analysis and helped to draft the results. All authors read and approved the final manuscript.

## Pre-publication history

The pre-publication history for this paper can be accessed here:

http://www.biomedcentral.com/1472-6963/10/66/prepub

## Supplementary Material

Additional file 1 **Patient questionnaire**. Questionnaire used to assess the willingness of patients identified as nonurgent to be reoriented to a hypothetical PCU outside the ED and to explore factors associated or not with this reorientation. Click here for file

Additional file 2 **ED physician questionnaire**. Questionnaire used to assess the ED visit. Click here for file
