# Supplementary material for: Nonurgent patients in the emergency department? A French formula to prevent misuse
Source: BMC Health Serv Res. 2010 Mar 15;10:66. doi: 10.1186/1472-6963-10-66 (PMC2846926; doi:10.1186/1472-6963-10-66)
Supplement: Additional file 1 — Patient questionnaire. Questionnaire used to assess the willingness of patients identified as nonurgent to be reoriented to a hypothetical PCU outside the ED and to explore factors associated or not with this reorientation. [file 1472-6963-10-66-S1.DOC]

**Additional file 1**

Title: Patient questionnaire

Description: Questionnaire used to assess the willingness of patients identified as nonurgent to be reoriented to a hypothetical PCU outside the ED and to explore factors associated or not with this reorientation

**PATIENT QUESTIONNAIRE**

ON ARRIVAL

1. Date of ED visit : _____/____/______
2. Day of the week: ………………………
3. Time of arrival : ……………………….
4. Presenting complaint: ………………………….…………

SOCIO-DEMOGRAPHIC AND SOCIO-ECONOMIC CHARACTERISTICS

1. Age : _________
2. Gender : ❑ Male ❑ Female
3. Place of birth ? …………………………….
4. Marital status?

- Living without a partner
- Living with a partner

1. Do you have children? ❑ Yes ❑ No
2. what is the postal code of your home  ? ……………………..
3. What is your level of education?

- More than basic education
- Basic education or less

1. What is your employment status? ❑ Employed ❑ Unemployed
2. If yes, what is your job ? …………………………………..

| 1. Currently, what is your medical insurance? | 1. What is your supplementary health insurance? |
| --- | --- |
| - None | - None |
| - « Sécurité sociale » *(French health insurance)* | - Private supplementary health insurance |
| - CMU *(French health insurance designed specifically to individuals and families with low incomes and resources)* | - Supplementary CMU *(French supplementary health insurance designed specifically to individuals and families with low incomes and resources)* |

1. How long have you lived in Marseilles and its outskirts ?

- Passing
- Less than 6 months
- Less than 2 years
- More than 2 years

USUAL SOURCE OF CARE

1. Currently, have you a regular source of care? ❑ Yes ❑ No
2. Where do you usually go for healthcare?

- To my doctor
- To any physician
- By self-medication (I take care of myself alone, I'm never sick ...)
- To the hospital
- Other

1. How many times have you consulted with your doctor during the past year?

Number of times: ………………………..

1. How many times did you go to the emergency department during the past year?

Number of times: ………………………..

THE REASONS FOR YOUR PRESENCE AT THE EMERGENCY DEPARTMENT

1. Today, what is your principal reason for attending the ED? __________________________________________________________________________________________________________________________________________________________
2. Did you contact a general practitioner for this complaint before coming? ❑ Yes ❑ No
3. How much time has elapsed between the beginning of the complaint that you have today and the decision to go to the emergency?

- One day
- Less than a week
- More than a week (note the period:……………….)

1. Who took the decision to send you to the emergency department?

- My general practitioner
- myself
- A member of my family
- My employer
- Other

1. What is the main reason that led you to consult with emergencies (multiple answers possible with ranking 1 to X):

- Because it is serious
- Because it hurts (very badly)
- Because I am very embarrassed
- Because I'm anxious , I ‘m afraid
- Because I'm in a hurry
- Because I think I need an x-ray, a suture
- Because my doctor was absent
- Lack of medical care
- Because it is the responsibility of the Emergency Department
- Because I need a certificate (administrative reasons)
- Not applicable (The patient does not choose to come)
- Other _________________ (1 word).

1. On an emergency scale from 1 to 20, what would you rate your current emergency:___/20

WILLINGNESS OF PATIENTS TO BE REORIENTED OUTSIDE THE ED

1. If after you arrived and explained your problem to the nurse at the reception you had been proposed to be reoriented (e) to a healthcare structure outside the emergency department, would you have agreed? ❑ Yes ❑ No

| ***If yes,***   1. Would you still agree if this shift implied going away from the hospital? | ***If no,***   1. Would you be willing to pay a surcharge to be treated in the emergency department? |
| --- | --- |
| ❑ Yes ❑ No | ❑ Yes ❑ No |
| 1. Wy ? (Key words) | 1. Wy ? (Key words) |

1. Would you still agree if this shift implied going away from the hospital at your expense?

❑ Yes ❑ No
