# Supplementary material for: Nonurgent patients in the emergency department? A French formula to prevent misuse
Source: BMC Health Serv Res. 2010 Mar 15;10:66. doi: 10.1186/1472-6963-10-66 (PMC2846926; doi:10.1186/1472-6963-10-66)
Supplement: Additional file 2 — ED physician questionnaire. Questionnaire used to assess the ED visit. [file 1472-6963-10-66-S2.DOC]

**Additional file 2**

Title: ED physician questionnaire

Description: Questionnaire used to assess the ED visit

**ED PHYSICIAN QUESTIONNAIRE**

ON ARRIVAL

1. Date of ED visit : _____/____/______
2. Time of arrival : ………………….
3. Presenting complaint : ……………………………….…………

ABOUT CONSULTATION IN THE ED

1. What was done to the patient during consultation, apart from clinical examination by the doctor?

- An X-ray picture
- Biological assessment
- Dressing
- Suture
- A cast or splint
- A strapping (tape for sprains)
- A drug delivery
- A medical certificate (MIC)
- A simple prescription
- Other: ……………………………….…………

1. Orientation doctor's diagnosis: ……………………………….…………
2. The patient could have waited more than 24 hours? ❑ Yes ❑ No
3. In this condition, was the use of ED essential for this patient? ❑ Yes ❑ No
4. At this time and in the context of the patient, was there an alternative to consultation at ED?

❑ Yes ❑ No
